# 28 SmartCHANGE: Promoting Healthy Lifestyle in Childhood with Support of AI

**DOI:** 10.1093/eurpub/ckae114.139

**Published:** 2024-09-26

**Authors:** Gregor Jurak

**Affiliations:** University Of Ljubljana, Ljubljana, Slovenia

## Abstract

Notwithstanding major improvements in the treatment of NCDs, primary prevention strategies that target healthy individuals are a more effective solution compared to prevention of adverse outcomes at early stages of the disease or treating a fully developed disease. Additionally, even though biological risk factors usually emerge in adulthood, childhood and adolescents are the ideal period for risk-lowering strategies based on behaviour changes.

Given that 80% of parents of inactive children wrongly consider their children to be sufficiently active, that existing risk calculation tools are used on adults, and that there is very little understanding about the appropriate level of specific behaviours even among health professionals (who are forced to rely on imperfect tools such as BMI), there is a deep need for tools to fight back against NCDs more effectively.

This is where SmartCHANGE comes in: the project’s goal is to develop trustworthy, AI-based decision-support tools that will help health professionals and citizens reduce long-term risk of NCDs by accurately assessing the risk of children and teens and promoting optimised risk-lowering strategies.

By engaging users right from the start of the application’s development through the process of co-creation and by applying machine learning to ethical datasets, the project will revolutionise health monitoring and wellness encouragement for youth, as well as preventing their risks of contracting diseases later in life.

At the final stage, developed solution, including applications, real-time lifestyle data collection, behaviour and health records data, AI prediction models and risk-lowering strategies will be tested in proof of concept study through four different settings.

In Finland, the project involves school nurses collaborating with 14-year-olds, leveraging the synergy of education and health.

In the Netherlands, we integrate pediatric primary care with schools for the 11 to 14 years-olds, fostering a holistic support system.

In Portugal, the focus is on family physicians working alongside 6 to 10-year-olds, recognizing the influence of family dynamics on health.

In Slovenia, a community-based approach engages various healthcare experts in connected continuum for 6 to 10-year-olds, highlighting collective responsibility.

We look forward to presenting our work, receiving feedback, and inviting collaboration from other participants in HEPA 2024.

